# First report of Serotype-1 Marek’s disease virus (MDV-1) with oncogenic form in backyard turkeys in Turkey: a molecular analysis study

**DOI:** 10.1186/s12917-021-03130-2

**Published:** 2022-01-12

**Authors:** Hasan Ongor, Necati Timurkaan, Hasan Abayli, Burak Karabulut, Hakan Kalender, Sukru Tonbak, Hatice Eroksuz, Burhan Çetinkaya

**Affiliations:** 1 Department of Microbiology, Faculty of Veterinary Medicine, Firat University, 23110 Elazig, Turkey; 2 Department of Pathology, Faculty of Veterinary Medicine, Firat University, Elazig, Turkey; 3 Department of Virology, Faculty of Veterinary Medicine, Firat University, Elazig, Turkey

**Keywords:** Turkeys, MDV-1, *MEQ*, *pp38*, *vIL-8*, 132-bp tandem repeats

## Abstract

**Background:**

Marek’s disease (MD) is a lymphoproliferative disease caused by Gallid alphaherpesvirus 2 (GaHV-2, MDV-1), which primarily affects chickens. However, the virus is also able to induce tumors and polyneuritis in turkeys, albeit less frequently than in chickens.

**Results:**

This is the first study in Turkey reporting the molecular characterization of a MDV-1 strain detected in a flock of backyard turkeys exhibiting visceral lymphoma. Here, *MEQ*, *vIL-8*, *pp38* and 132-bp tandem repeat regions, which are frequently preferred in the pathotyping of MDV-1, were examined. It was determined that the *MEQ* gene of MDV-1/TR-21/turkey strain obtained in the present study encoded 339 amino acids (1020 nt) and had four proline-rich repeat regions (PPPP). Based on the nucleotide sequence of the *MEQ* gene of the MDV-1/TR-21/turkey strain, a phylogenetic tree was created using the MEGA-X software with the Maximum Likelihood Method (in 1000 replicates). Our strain was highly identical (> 99.8) to the Italian/Ck/625/16, Polish (Polen5) and some Turkish (Layer-GaHV-2-02-TR-2017, Tr/MDV-1/19) MDV-1 strains. Also, nt and aa sequences of the *MEQ* gene of our strain were 99.1 and 99.41% identical to another Turkish strain (MDV/Tur/2019) originated from chickens. Sequence analysis of *pp38* and *vIL-8* genes also supported the above finding. The identity ratios of nucleotide and amino acid sequences of *vIL-8* and *pp38* genes of MDV-1/TR-21/turkey strain were 99.64–100% and 99.79–100%, respectively, when compared with those of the Polish strain. According to 132-bp tandem repeat PCR results, the MDV-1/TR-21/turkey strain had five copies.

**Conclusions:**

These results suggested that the MDV-1/TR-21/turkey strain obtained from backyard turkeys can be either very virulent or very virulent plus pathotype, though experimental inoculation is required for precise pathotyping.

## Background

Marek’s disease (MD) is a common lymphoproliferative and neuropathic disease of chickens, and occasionally of turkeys, quails and geese caused by gallid alphaherpesvirus 2 (GaHV-2). However, reports of MD in turkeys are increasing worldwide in the last few years. The etiological agent, commonly known as Marek’s disease virus (MDV) is a member of the genus *Mardivirus*, sub-family *Alfaherpesvirinae* in family *Herpesviridae*. MDVs are classified into three different species which correspond to previously described serotypes: GaHV-2 (serotype 1- MDV-1), GaHV-3 (serotype 2- MDV-2) and meleagrid alphaherpesvirus 1 (MeHV-1) or herpesvirus of turkeys (HVT) (serotype 3) [1–3]. There is also an additional classification for serotype 1/MDV-1 that divides the viruses into four pathotypes that is based on virulence in chickens in vaccination challenge studies: the mild (m), virulent (v), very virulent (vv) and very virulent plus (vv+) forms [4]. This method is time consuming, expensive and requires many chickens of a specific genetic type and MD-antibody status. Serotype 1 (MDV-1) includes all the virulent strains and some attenuated vaccine strains and, only viruses in this serotype are able to cause the disease in chickens.

MDV is oncogenic in chickens and clinical signs include immunosuppression, polyneuritis and T-cell lymphoma formation in the visceral and ectoderm-derived tissues. Although the virus can also induce tumors in turkeys, more frequently detected oncogenic agents in this species include the reticuloendotheliosis virus (REV), avian sarcoma leukosis virus (ASLV) and the lymphoproliferative disease virus (LPDV) MD is a major threat for the poultry industry because of the economic consequences in the absence of efficient control strategies [1]. Vaccination with non-oncogenic HVT, MDV serotype 2 (MDV-2) or attenuated MDV-1 vaccines is frequently used for the control of the disease in chickens [5]. On the other hand, turkeys are rarely vaccinated against MD. Although MD vaccines have successfully been used in reducing major losses due to the disease, several factors such as improper use of the vaccine, exposure to virulent viruses before the development of immunity, interference from maternally derived antibodies and emergence of new virulent strains have limited the effectiveness of the vaccines. In fact, MDV has evolved into a more virulent form and the emerging strains not only are capable of breaking vaccine immunity but also show immunosuppressive effects [6, 7].

The MDV genome contains more than 200 genes and among these genes, Marek’s EcoRI-Q (*MEQ*), phosphoprotein-38 (*pp38*) and viral interleukin 8 (*vIL-8*) have been reported to play important role in the virulence of MDV-1 [7, 8]. The *MEQ* gene encodes a 339-amino acid protein with an N-terminal basic region leucine zipper (bZIP) domain and a C-terminal transactivation domain. The bZIP domain, similar to that of the Jun/Fos family of oncoprotein, consists of two stretches of basic residues basic regions 1 and 2 (BR1 and BR2) and a leucine zipper. The transactivation domain is characterized by 2.5 proline-rich repeats (PRRs), which contain several SH3-binding motifs [9]. The *MEQ* gene is regarded as one of the principle oncogene of MDV-1 and also contributes to immunosuppression. This gene plays a key role in the transformation process of latently-infected T lymphocytes. Since MDV-2 and HVT viruses do not carry the *MEQ* gene, they are considered as avirulent and generally used in the preparation of effective vaccines [10]. The *pp38* gene is responsible for inhibiting the maturation of lymphocytes and for cytolytic replication [3]. The *vIL-8* gene, a viral chemokine, ensures early cytolytic infection and latency [7].

BamHI-H is a transcriptionally active region [11], and it was suggested that the loss of oncogenicity associated with 132-bp expansion could be due to a direct effect on the BamHI-H gene family transcripts. When MDV is attenuated by continuous cell culture, the copy number of 132-bp repeats in BamHI-H region often increases from two to more than 20 copies [12]. Therefore, the 132-bp of the MDV-1 genome can also be used as an important PCR target gene to differentiate the field MDV strain from the vaccine strain [13].

Various methods such as virus isolation, pathology and immunohistochemistry that have advantages and disadvantages over each other have been used in the diagnosis of MD. By the means of molecular based methods such as PCR and real–time PCR (qPCR), it is possible to obtain a good indication of pathotype/virulence of the virus owing to the amplification of oncogenicity/pathogenicity related genes followed by sequencing. This also enables us to better understand why protection is not provided in some vaccinated flocks and then develop more efficient vaccines containing the correct pathotype based on molecular characterization [14]. The PCR analysis of the BamH1-H and BamH1-D regions of the viral genome flanking the 132-bp tandem repeat region is an important criterion that can be used to distinguish between pathogenic and non-pathogenic strains of MDV-1. Although there is a paucity of information on the relationship between the number of 132-bp tandem repeats and pathogenicity of virus strains in turkeys, it has been reported that only one or two repeats of the 132-bp region were present in the most pathogenic strains of MDV in chickens [5].

This study was carried out to investigate the molecular characteristics of *MEQ, pp38, vIL-8* genes and BamH1-H and BamH1-D regions of the viral genome flanking the 132-bp tandem repeat region in the field strain originated from visceral tumors and feather tips of turkeys by polymerase chain reaction (PCR). In addition, the amplified products were subjected to sequencing in order to determine pathotype of the field strain and construct a phylogenetic tree.

## Results

### Gross and histopathological findings

**Fig. 1:**
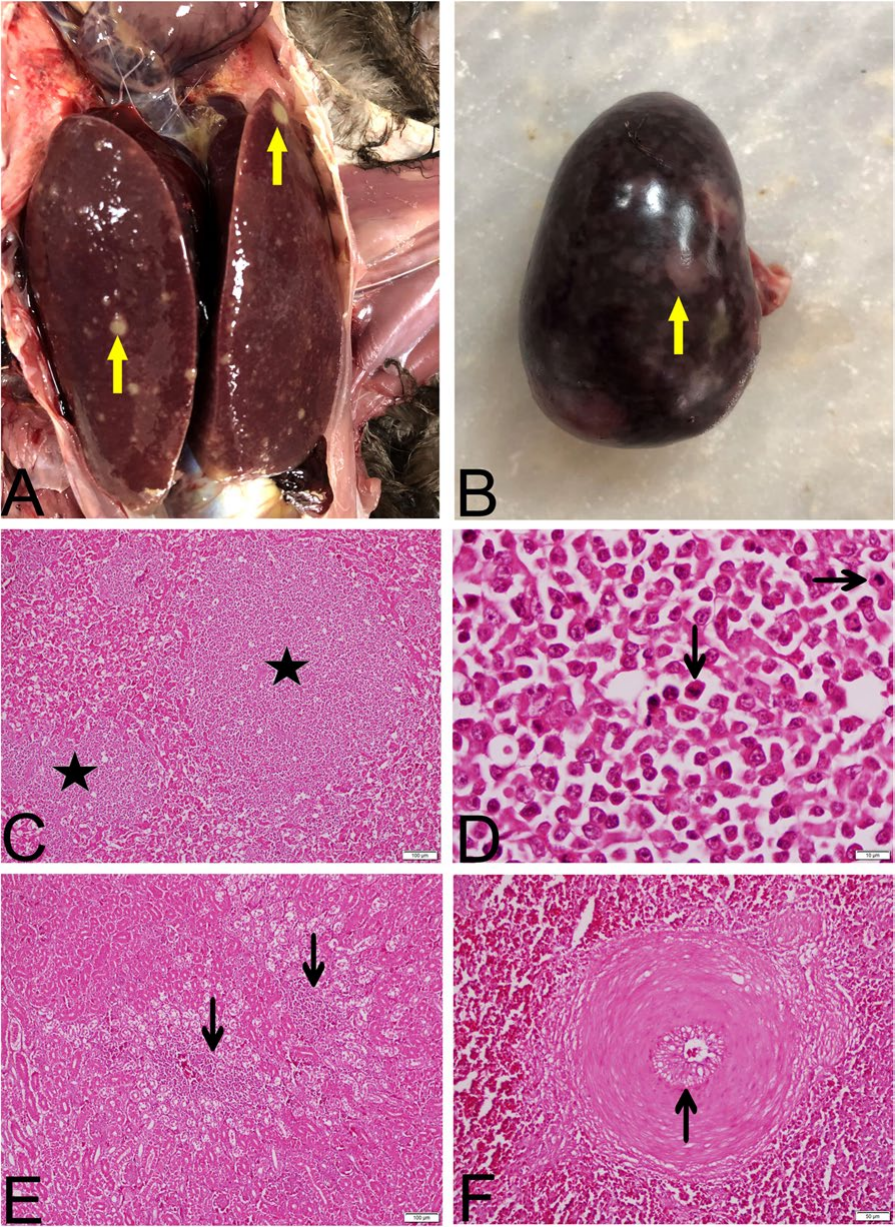
Liver **(A)** and spleen **(B)** enlarged and multifocal nodules of varying size (arrows). Liver sections **(C and D)** showing nodular structures (stars) consisting of atypical and pleomorphic lymphoid cells with numerous mitotic figures (arrows) in hepatic parenchyma and decreasing amount of hepatocytes. Kidney section **(E)**, tumor cells (arrows) between the renal tubules and glomeruli. Spleen section **(F),** an artery showing atherosclerosis (arrow). (**C**: 100X, **D**:1000X, **E**:100X, **F**:400X)

Liver and spleen were enlarged in both animals necropsied, with multifocal, nodular structures of varying sizes (Fig. 1A, B). It was also noted that the glandular stomach was thickened in one of the turkeys as well as enlargement in the regional lymphoid structures of the intestines.

In the histopathological examination, pleomorphic lymphoproliferative foci of varying size were observed in the liver (Fig. 1C), spleen, lungs (in both animals), heart, kidneys, proventriculus, and intestines (in one animal). These cells were detected to be atypical and, pleomorphic lymphoblasts and lymphocytes. The tumor cells displayed marked mitotic figures (> 8 per high-power fields) (Fig. 1D). Tumor cells were found to be located multifocally in areas, ranging from small foci to nodular structures in the liver, lungs and spleen. In the liver, tumor cells were also found intensively in the sinusoids. Tumor cells were located especially in the portal areas in one of the turkeys. It was observed that the normal histological structure of the liver was impaired and the number of hepatocytes was considerably reduced. Tumor cells were found between the muscle fibers in the heart, especially around the veins, between the renal tubules and glomeruli in the kidney (Fig. 1E) and in the propria mucosa in the proventriculus and intestines. Mild edema and lymphocytic infiltration were noticed in the ischiadic nerve in both animals, but no tumor cells were observed. In addition, atherosclerosis characterized with arterial lipid accumulation in the intima and luminal narrowing was detected in the arteries of the spleen in one case (Fig. 1F).

### Analysis of the MEQ gene

Following PCR combined with *MEQ* primers, the amplification product of approximately 1050 bp in length was detected on the agarose gel. As a result of bidirectional sequencing of the purified PCR product, the *MEQ* gene (1020 nt in length) encoding 339 aa was detected. According to BLAST search, the nucleotide sequence of the *MEQ* gene was similar (up to 99.9%) to the MDV-1 strains in the GenBank. The strain obtained in the study was named as MDV-1/TR-21/turkey and the *MEQ* gene sequence was submitted to the GenBank Nucleotide Sequence Database under the accession number OK322357. Table 1 presents detailed information on MDV-1 strains selected from GenBank with different pathotypes originated from different regions. MDV-1/TR-21/turkey strain and some Turkish (Layer-GaHV-2-02-TR-2017 and TrMDV-1/19), Italian (GaHV-2/Italy/Turkey/601/16 and GaHV-2/Italy) The /Ck/625/16), and Polish (Polen5) MDV-1 strains were in the same cluster in the phylogenetic tree, which were highly identical (> 99.8%). The nt and aa sequences of the *MEQ* gene of our strain were 99.1 and 99.41% identical to another Turkish strain originated from chickens (MDV/Tur/2019), while 99.93 and 99.76% similarities were determined between our strain and the Italian turkey strain (GaHV-2/Italy/Turkey/601/16), respectively.

**Table 1:** Details of the MDV-1 strains, retrieved from GenBank, which were used for the phylogenetic analysis

GaHV-2 strain	Country	Year	Pathotype	Size	PPPPs	Host	Accession number	References
CVI988	Netherlands	1969	Att	398	7	NA	DQ530348	[15]
814	China	1986	att	398	7	Chicken	JF742597	[16]
3004	Russia	NA	att	398	7	NA	EU032468	NA
CU-2	USA	1970	m	398	7	*Gallus gallus*	AY362708	[17]
MD70/13	Hungary	1970	v	339	5	*Gallus gallus*	MF431495	[18]
617A	USA	1993	v	339	4	NA	AY362712	[17]
04CRE	Australia	2004	v	398	5	NA	EF523773	[19]
EU-1	Italy	1992	vv	339	5	*Gallus gallus*	MF431494	[18]
MD5	USA	1977	vv	339	4	NA	AF243438	[7]
Woodlands1	Australia	1992	vv	339	5	NA	EF523775	[19]
RB1B	USA	NA	vv	339	5	NA	AY243332	[17]
648A	USA	1994	vv+	339	2	NA	AY362725	[17]
New	USA	1999	vv+	339	2	NA	AY362719	[17]
W	USA	1999	vv+	339	4	NA	AY362723	[17]
ATE2539	Hungary	2000	vv+	339	5	*Gallus gallus*	MF431493	[18]
Polen5	Poland	2010	vv+	339	4	*Gallus gallus*	MF431496	[20]
Tn-n1	India	2012	NA	339	5	*Gallus gallus* domesticus	HM749324	NA
UDEACO	Colombia	2013	NA	339	2	*Gallus gallus* breed layer	KU058701	[21]
GaHV-2/Italy/Ck/507/15	Italy	2015	NA	418	9	backyard chicken	MK139661	[22]
GaHV-2/Italy/Ck/625/16	Italy	2016	NA	339	4	backyard chicken	MK139666	[22]
Layer-GaHV-2-02-TR-2017	Turkey	2017	NA	339	4	layer chicken	MN045205	[23]
MDV/Tur/2019	Turkey	2019	High oncogenity	339	5	cochin chicken	MN956505	[24]
TrMDV1/19	Turkey	2019	NA	339	4	commercial chicken	MN817545	[25]
GaHV-2/Italy/Turkey/601/16	Italia	2016	NA	339	4	meat type turkey	MN017102	[22]
MDV-1/TR-21/turkey	Turkey	2021	–	339	4	backyard turkey	OK322357	in this study

The *MEQ* of our strain was detected to have four proline-rich repeat regions (PPPP). The start of PPPPs in the *MEQ* gene was based on amino acid positions 152, 175, 191 and 232. It was found that another PPPP starting at position 216 of the *MEQ* was disrupted due to substitution at amino acid position 218 (P to S). The amino acid substitutions detected between the *MEQ*s of MDV-1/TR-21/turkey and some other MDV-1 strains after multiple alignment are shown in Table 2. The aa substitutions of the *MEQ* of MDV-1/TR-21/turkey were as follows: 77 (K to E), 80 (D to Y), 88 (T to A), 93 (R to Q), 110 (C to S), 115 (L to V), 119 (R to C), 139 (A to T), 153 (Q to P), 176 (A to P), 180 (A to T), 217 (A to P), 218 (P to S), 277 (P to L), 283 (V to A), 315 (S to W) and 320 (T/N to I).

**Table 2:** Amino acid substitutions in *MEQ* oncoprotein of the Turkish strains and the reference strains

(Pathotypes) Strain/isolates	Basic region		Leucine Zipper					Repression/Transactivation domain									
	77	80	88	93	110	115	119	139	153 PPPP	176 PPPP	180	217	218 PPPP	277	283	315	320
MDV-1/TR-21/turkey	E	Y	A	Q	S	V	C	T	P	P	T	P	S	L	A	W	I
(v)MD70/13	K	D	.	.	C	.	.	.	.	.	.	.	P	.	.	S	.
(v)617A	.	.	.	.	C	.	R	.	.	.	.	A	P	.	.	S	.
(vv)Md5	K	D	.	.	C	.	.	.	.	.	.	A	P	.	V	S	T
(vv)RB1B	K	D	.	.	C	.	.	.	.	.	.	.	P	.	.	S	.
(vv+)648A	K	D	.	.	C	.	R	.	Q	A	A	A	P	P	.	S	.
(vv+)New	K	D	.	.	C	.	R	.	Q	A	.	A	P	.	V	S	T
(vv+)W	K	D	.	.	C	.	.	.	.	.	.	A	P	.	V	S	T
(vv+)ATE2539	.	.	T	R	C	.	.	A	.	.	.	.	P	.	.	S	.
(vv+)Polen5	.	.	.	.	.	.	.	.	.	.	.	.	.	.	.	S	.
GaHV-2/Italy/Ck/625/16	.	.	.	.	.	.	.	.	.	.	.	.	.	.	.	S	.
Layer-GaHV-2-02-TR-2017	.	.	.	.	.	.	.	.	.	.	.	.	.	.	.	–	–
MDV/Tur/2019	.	.	T	R	C	L	.	A	.	.	.	.	P	.	.	S	.
TrMDV1/19	.	.	.	.	.	.	.	.	.	.	.	.	.	.	.	S	N
GaHV-2/Italy/Turkey/601/16	.	.	.	.	.	.	.	.	.	.	.	.	.	.	.	S	.

Pathotypes of virulent MDV; vMDV, very virulent MDV; vvMDV, very virulent plus MDV; vv + MDV

“.” mean same to the consensus amino acids below corresponding sites

“-” mean lacking of consensus amino acids below corresponding sites

### Analysis of the *vIL*-8 and *pp38* genes

Following PCR combined with primers specific for *vIL-8* and *pp38* gene, amplification products at approximately 900 bp and 1000 bp lengths were obtained, respectively. Of these, 887 and 1006 bp lengths were associated with MDV-1 in the BLAST search. The *vIL-8* gene of MDV-1/TR-21/turkey was highly identical (nt 99.65%, aa 100%) to Chinese (mg432607), Polish (Polen5), Italian (EU-1) and Hungarian ATE2539 strains. Similarly, the *pp38* gene of the MDV-1/TR-21/turkey strain was highly identical (nt 99.7%, aa 100%) to Italian (EU-1), Turkish (MDV/Tur/2019), Polish (Polen5), Hungarian (MD70/13 and ATE2539), American (CU-2) and Chinese (MG432697 and 814) strains.

The sequence data of the *vIL-8* and pp38 genes of the MDV-1/TR-21/turkey strain were submitted to the GenBank Nucleotide Sequence Database under the accession numbers OK322358 and OK322359, respectively.

### Analysis of 132-bp tandem repeats

Following PCR combined with primers specific for 132-bp tandem repeats, 830 bp amplification product was obtained on agarose gel. According to the results, the MDV-1/TR-21/turkey strain had five copies of the 132-bp tandem repeats.

**Fig. 2:**
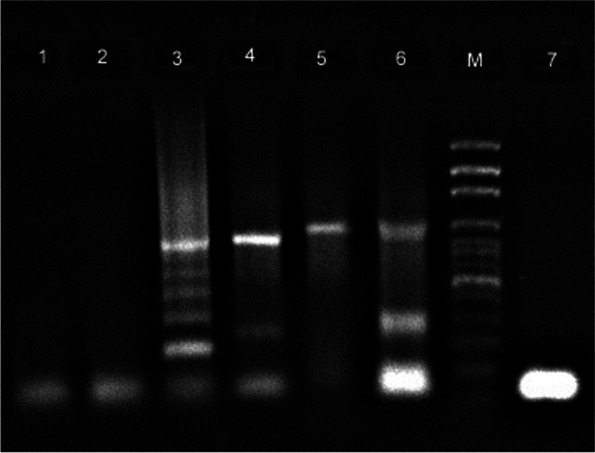
Agarose gel electrophoresis (1.5 w/v) of PCR products of MDV-1 and REV. M; 100 bp DNA ladder (Solis BioDyne, USA). Lane 1–2; Negative controls for MDV and REV, respectively, PCR amplification of 132-bp tandem repeats (Lane-3; 830 bp), *pp38* (Lane 4; 887 bp), *vIL-8* (Lane-5; 1006 bp), *MEQ* (Lane-6; 1020 bp), and REV (Lane-7)

Agarose gel analysis of PCR amplification results for *MEQ*, *pp38*, *vIL-8*, and 132-bp tandem repeats of MDV-1 and REV was shown in Fig. 2. No positive results were obtained in the PCR analysis for REV.

## Discussion

In spite of the fact that MD is rarely seen in turkeys, it has been reported in some countries including Netherlands, France, the UK, Israel, Germany, Scotland, Egypt and the USA [14, 22, 26, 27]. Severe outbreaks of the disease have been reported in commercial turkey flocks in Europe and Israel between 1997 and 2002 [1, 28]. In some of these outbreaks, the affected turkey flocks were observed to be raised in proximity to broiler chickens. High mortality rates ranging from 40 to 80% have been noted in some countries [1, 28]. In the USA, the presence of MD in turkeys was first reported by Hauck et al. [29] who detected MDV-1 in house-type and zoo-raised turkeys based on both immunohistochemical and molecular methods. In Turkey, first cases of MD in turkeys have been reported in 2020 based on pathological and immunohistochemical analyses [30]. The turkey is the natural host of HVT and the virus can circulate in turkey populations without causing disease [31]. The fact that turkeys are not frequently infected with MDV has been explained with genetic resistance and widespread presence of HVT which could provide some protection against the disease. However, detection of highly oncogenic MDV-1 in HVT-vaccinated turkeys in an experimental study casts doubt on this view [32].

Gross and histopathological findings observed in the current study were mostly compatible with previously reported MD cases in avian species [1, 30]. As an interesting finding, atherosclerosis that was detected in an artery of spleen in one turkey has also been noted in both natural and experimental MD infections of chickens [33–35], though its pathogenesis is yet to be clarified. In a study conducted by Reddy et al. [27], very virulent MDV strain was detected in the cytological and histopathological examination of lymphomas in the internal organs of vaccinated turkey flocks, though the type of the vaccine was not indicated. It has therefore been reported that vaccination of turkeys may not protect them against increasingly virulent field strains of MDV [27]. However, it should be underlined that false positive results might have been obtained from cytological and histopathological examinations, and detection of the pathotype by using molecular tools can produce more accurate results [36]. It was suggested that apart from in vivo pathotyping assays on susceptible chickens [4], molecular sequencing is one of the most suitable method for exact classification of MDV-1 strains.

The nucleotide-based classification of MDV virulence based on their *MEQ* gene is highly advantageous when compared to the classical gold standard method of pathotype determination. The great divergence of the *MEQ* gene sequences enabled Shamblin et al. [17] to characterize distinctive polymorphisms and point mutations that correlated with the virus virulence. Previous reports have suggested that the most virulent MDV strains contain the lowest (2–5) number of PPPP repeats in the *MEQ* gene, while the low-pathogenicity and/or attenuated MDV strains show the highest (7–8) number of PPPP repeats [17, 19, 22]. In the sequence analysis of the *MEQ* gene, four PPPP repeats were detected in the present study. In addition, the detection of a disruption at one of the PPPP repeats due to substitution at amino acid position 218 (P to S) suggested that the MDV-1 strain obtained here could belong to vv or vv + pathotype.

The *MEQ* gene of MDV-1/TR-21/turkey was highly identical to the Italian strain GaHV-2/Italy/Turkey/601/16, but unlike the Italian strain, our strain had an amino acid substitution at position 315 (S to W) of the *MEQ*. The MDV-1/TR-21/turkey strain and other MDV-1 strains reported in commercial and layer chickens in Turkey were also similar in character and contain several aa substitutions in the *MEQ* gene (S315W and N320I) [23–25]. Our strain was 99.8% identical to another Turkish Layer-GaHV-2-02-TR-2017 strain based on the data of partial *MEQ*. Controversial opinions have been put forward about horizontal transmission between turkeys and chickens. Although some MD cases in commercial turkey flocks have been linked with the contamination originated from chickens, it has been reported that the epidemiological role of chickens might be limited [22, 28, 29, 37]. In this study, the high genetic similarity detected between our turkey strain and other strains derived from chickens may be an important indicator for interspecies transmission. On the other hand, the turkeys sampled here were originated from a house type flock which also included chickens, and although there was no information about the vaccination history in this flocks, no disease was observed in the chickens. In this case, it is plausible to suggest that our MDV strain adapted to turkeys. All in all, large scaled studies are needed to have a better understanding of the epidemiology of MDV-1 infection in turkeys.

In addition to *MEQ* gene, molecular investigation of other virulence associated markers such as *pp38* and *vIL-8* plays important role in the detection of pathotype of MDV-1. The results of *vIL-8* gene and *pp38* gene analysis were consistent with those of *MEQ* and showed high similarity to Polish (Polen5), Italian (EU-1) and Hungarian ATE2539 strains. Some researchers have previously reported that the repeat numbers of BamH1-H and BamH1-D may also be important in the pathotyping of MDV-1 [5, 25]. The 132-bp repeat numbers have been reported to be only one or two in the most pathogenic strains of MDV-1, while six or seven in mild pathogenic strains in chickens [21]. In this study, we found that our strain had five copies of 132-bp tandem repeats. Although this seems to conflict with the previous reports for *MEQ* analysis which is frequently used for pathotyping, it is not possible to make a precise comparison because no data are available for 132-bp tandem repeats in turkeys. Also, the number of 132-bp tandem repeats may not have an absolute link to virulence.

In the last decade, the vv + pathotype of MDV-1 has been reported to be predominant [16, 17, 38] that may explain why the disease occurred in vaccinated chicken flocks. Previous studies conducted in our country have also revealed the presence of MDV-1 field isolates (pathotype vv+) in vaccinated chickens with disease manifestations [24, 25]. Very virulent (vv) and vv + pathotypes of MDV-1 frequently induce higher mortality and more visceral lymphomas, and have the tendency to more frequently break through genetic host resistance or immunity induced by vaccination.

The newly emerging variants and increase in oncogenicity/pathogenicity of MDV due to the consistent changes in the virus cause significant economic losses in poultry. This feature of the MDV may also lead to the insufficient protection of the vaccines. Today, field strains of MDV have been shown to be highly infectious and might cause disease in vaccinated flocks [23, 25]. It has been reported that HVT vaccine did not show protection against MDV-1 virulent pathotypes in turkeys, but vaccination with prototype CVI988 vaccine (Rispens) has successfully been used against these pathotypes in France and Switzerland [28]. However, some studies failed to show this protection [32]. It is therefore crucial to develop more effective vaccines against the disease.

## Conclusions

Although the number of MD cases in turkeys is increasing in the world, there is a paucity of sequence data for MDV-1 in this species in the GenBank. The current study was the first in our country that indicated the pathotype of the virus isolated from turkeys by detecting the significant markers using molecularV tools. *MEQ*, *pp38* and *vIL-8* gene data of MDV-1/TR-21/turkey strain obtained from backyard turkeys here will contribute to the knowledge on host distribution and molecular epidemiology of MDV-1. It was concluded that vaccine selections should be based on the determination of pathotype of the field MDV-1 isolates by molecular tools. Although vaccination against MD is applied regularly in chickens due to significant economic losses, it is not widely used in turkeys because of low incidence. The results of the present study suggested that vaccination in turkeys against MDV, which is oncogenic and highly virulent, could be considered in both Turkey and other countries as well as good management practices and efficient biosafety measures.

## Materials and methods

### Sample history and processing

Two dead six-month-old turkeys (Bourbon red strain) from a flock with the complaints of loss of appetite, inability to stand up, diarrhea and death within 1 week were submitted to the Veterinary Faculty of Firat University located in the east of Turkey. The turkeys belonged to a small family type flock (the capacity was 30 animals in total) which also included chickens. However, no disease symptoms were noted in chickens. Systemic necropsy was performed, and tissue (liver, spleen, lungs, heart, kidneys, brain, eyes, proventriculus and intestines) specimens were fixed in the 10% neutral formalin solution, and placed in standard tissue processing cassettes. After routine tissue processing and paraffin embedding procedures, histological sections were taken from the paraffin blocks with a rotary microtome, stained with hematoxylin eosin, and examined under light microscopy.

### Virological analysis

Tumor-bearing organs of turkeys collected at necropsy were aliquoted for virological examinations and stored at − 20 °C. Sections from tumor-bearing organs (liver and kidney) and feather tips were homogenized in 1X phosphate-buffered saline (PBS, pH 7.2–7.4). The resulting homogenate was centrifuged at 1500 x g for 15 min. DNA-RNA isolation from the supernatant was performed with the QIAamp MinElute Virus Spin kit (Qiagen, Hildeni Germany) according to the manufacturer’s instructions. Extracted nucleic acids were eluted in 50 μL of elution buffer, quantified in a spectrophotometer (Nanodrop 2000, Thermo Fisher, MA, USA) and kept at − 20 °C until analysis.

### Detection of REV and MDV

For the detection of REV, we followed the polymerase chain reaction (PCR) procedure described by Ongor and Bulut [39]. Specific *MEQ* [23] and 132-bp repeat primers [13] were used to detect MDV, another oncogenic virus. The DNA template was then used for amplification of the *pp38* and *vIL-8* regions of MDV [13]. The PCR procedure applied in the reference for diagnosis and identification of MDV was followed without modification. The obtained amplicons were analyzed by electrophoresis (110 V/40 min) in 1.5% (w/v) agarose-TAE (40 mM Tris–acetate, pH 8.0, 1 mM EDTA) gel stained with ethidium bromide. The sequences of primer sets selected for this study are shown in Table 3.

**Table 3:** Primers used for detection and identification of MDV and REV

Virus/Gene	Primer sequence (5′-3′)	Amplicon size	References
*MEQ*	ATGTCTCAGGAGCCAGAGCCGGCGCT GGGGCATAGACGATGTGCTGCTGAG	1062	[40]
		(1242)	
*pp38*	TCATCTTCAACCCACAGCCATCC TCGCTTAATCTCCGCCTCCAAC	1006	[13]
*vIL-8*	GAGACCCAATAACAGGGAAATC TAGACCGTATCCCTGCTCCATC	887	
132-bp repeat	ATGCGATGAAAGTGCTATGGAG ATCCCTATGAGAAAGCGCTTGA	Variable	
REV	GAAGCAGACAATAGGACTGG TTGACCTAGGGTATCCATCTC	850	[39]

### Sequencing and phylogenetic analysis

**Fig. 3:**
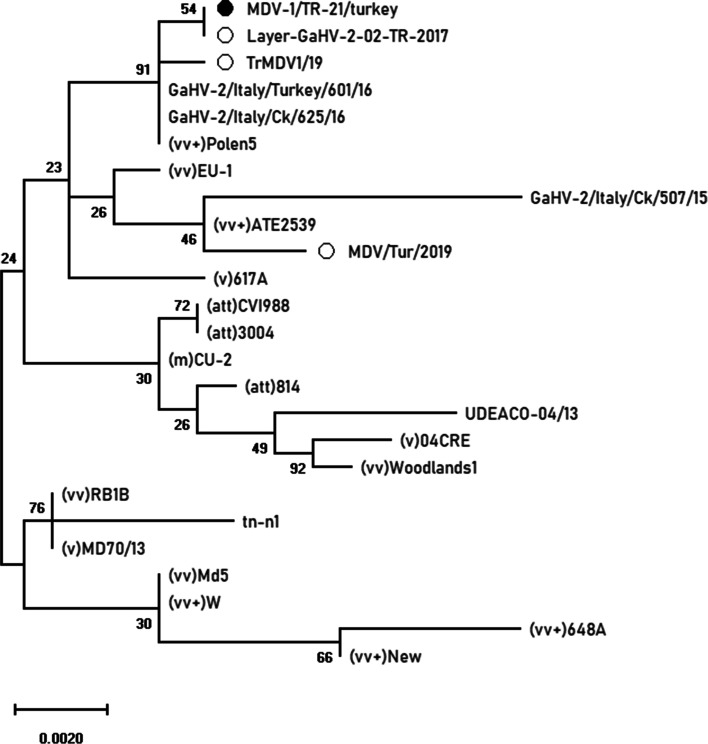
Phylogenetic analysis of the *MEQ* gene of MDVs. Phylogenetic trees were created using the Maximum Likelihood method (1000 replicates) and Tamura Nei model by the Mega X. Filled circles represent the Turkish MDV strain obtained from this study, while the unfilled circles represent other Turkish MDV strains

**Fig. 4:**
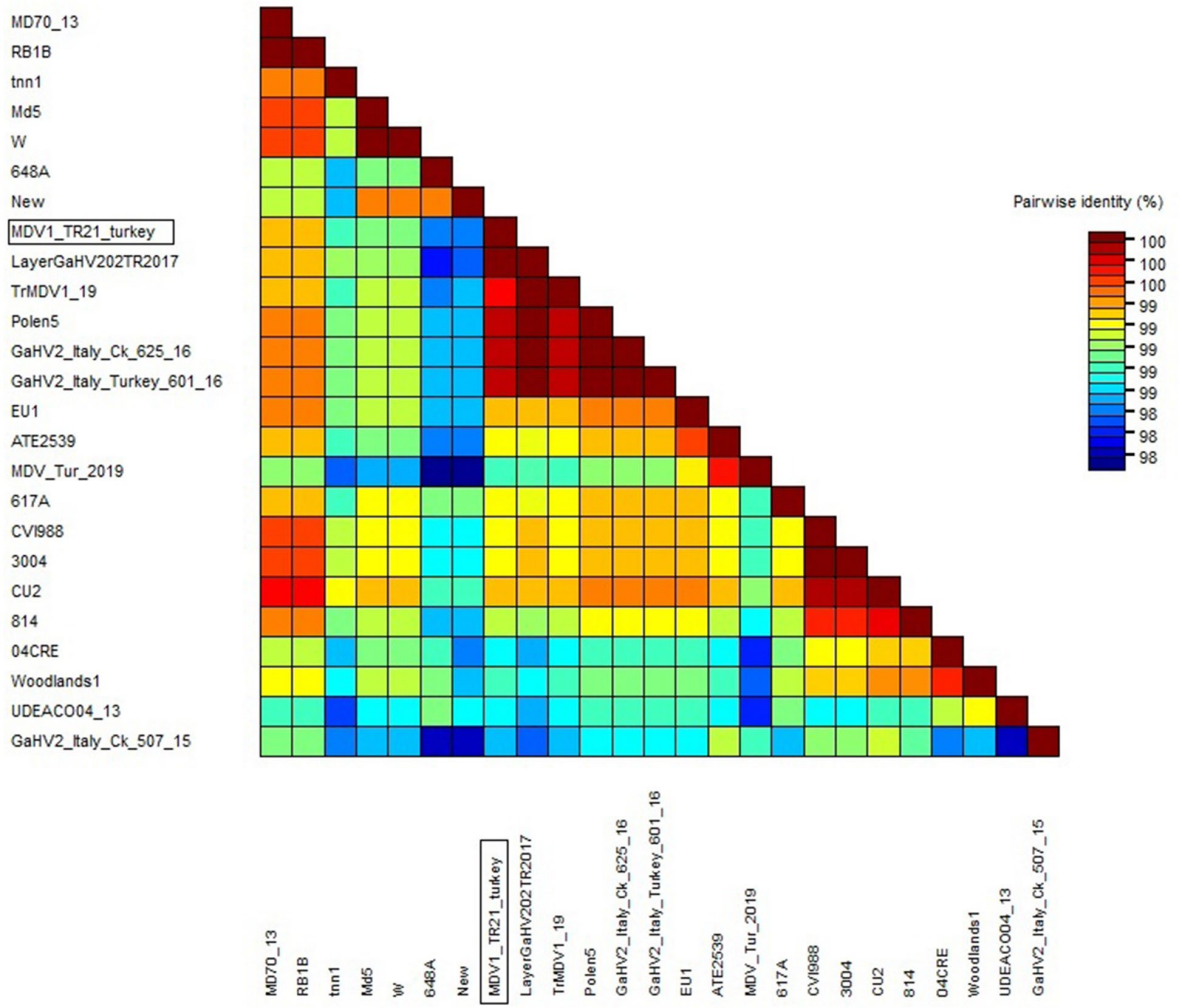
Pairwise identity analysis on nucleotide sequencing of the *MEQ* gene of MDV strains obtained from this study and selected from GenBank

PCR products were gel purified and sequenced in a commercial sequencing service (Macrogen Europe, Amsterdam, The Netherlands). This stage was performed in an ABI Prism 3130 genetic analyzer (Applied Biosystems), using the BigDye Terminator v3.1 cycle sequencing kit (ApApplied Biosystems). Bidirectional nucleotide sequences aligned by Clustal W software was edited, verified with BLAST (BLAST: Basic Local Alignment Search Tool (nih.gov) and submitted to the GenBank database. Then, nucleotide sequence data of MDV strain obtained from this study and other strains selected from GenBank were transferred to Mega X software [41]. Aligned nucleotide sequences were converted to amino acids and compared with each other. Also, using the aligned nucleotide sequences, a phylogenetic tree for *MEQ* gene was constructed with MEGA X software (Fig. 3). At this stage, Maximum Likelihood method and Tamura Nei model with 1000 bootstrap replicates were used [42]. The pairwise identity of the multiple aligned sequences was calculated using Sequence Demarcation Tool Version 1.2 (SDTv1.2) [43] and the graph was drawn according to the phylogenetics generated by the Neighbor joining method. The MDV-1 strains in Table 1 were used when constructing the phylogenetic tree and calculating sequence pairwise identity (Fig. 4).

## Data Availability

The datasets used and/or analyzed during the current study are available from the corresponding author on reasonable request.

## Abbreviations

MD: Marek’s disease
MDV: Marek’s disease virus
GaHV: Gallid alphaherpesvirus
ASLV: Avian sarcoma leucosis virus
HVT: Turkey herpesvirus
REV: Reticuloendotheliosis virus
LPDV: Lymphoproliferative disease virus
*MEQ*: Marek’s EcoRI-Q-encoded protein
*PP38*: Phosphoprotein-38
*vIL*-8: Viral interleukin 8
PPPP: Four proline molecules
